# Transcatheter Edge-to-Edge Repair of Tricuspid Regurgitation With TriClip: A Step-by-Step Guide

**DOI:** 10.31083/RCM44502

**Published:** 2025-11-18

**Authors:** Georgios E. Papadopoulos, Ilias Ninios, Sotirios Evangelou, Andreas Ioannides, Grigorios Giamouzis, Vlasis Ninios

**Affiliations:** ^1^Cardiology Department, Interbalkan Medical Center, 57001 Thessaloniki, Greece; ^2^Department of Cardiology, University Hospital of Larissa, 41334 Larissa, Greece

**Keywords:** tricuspid regurgitation, transcatheter edge-to-edge repair, TriClip system, structural heart disease, intraprocedural imaging, transesophageal echocardiography, tricuspid valve intervention, PASCAL system, combined mitral and tricuspid repair

## Abstract

Tricuspid regurgitation (TR), a condition that was once thought to be of little clinical importance, is now recognized as a progressive disease associated with increased morbidity and mortality. Despite the prevalence of TR, this condition remains undertreated due to the absence of effective medical therapy and high surgical risk. However, tricuspid transcatheter edge-to-edge repair (T-TEER) using the TriClip system has emerged as a new approach, offering a minimally invasive alternative for patients with symptomatic severe TR and prohibitive surgical risk. Thus, this comprehensive review outlines a step-by-step approach to TriClip implantation, encompassing anatomical and pathophysiological foundations, patient selection criteria, imaging protocols, and procedural techniques. Emphasis is placed on the critical role of transesophageal echocardiography for device guidance, leaflet grasping, and confirmation of procedural success. Moreover, key intra-procedural challenges and troubleshooting strategies are discussed in detail, along with post-procedural management, including antithrombotic therapy, imaging surveillance, and functional assessment. Comparative insights between TriClip and the PASCAL system are provided, highlighting technical and clinical differences, as well as implications for device selection. The emerging role of combined mitral and tricuspid TEER using a single steerable guide catheter is also explored, supported by early data suggesting the safety and efficacy of this combination. Evidence from randomized trials and real-world registries supports the safety, feasibility, and durability of TriClip-based T-TEER. Notably, as experience and technology continue to evolve, T-TEER is positioned to become a cornerstone in the management of functional TR in high-risk populations.

## 1. Introduction 

Tricuspid regurgitation (TR) has emerged as a significant clinical entity associated with increased cardiovascular morbidity and mortality [1]. Long considered a benign bystander known as the “forgotten” valve, moderate-to-severe TR is now known to affect millions globally, with increasing prevalence among elderly patients and women [2, 3, 4]. Despite its clinical significance, TR remains undertreated: guideline-directed medical therapies lack Class I recommendations [5, 6], and isolated tricuspid valve surgery carries a prohibitive risk since patients are often diagnosed at an advanced stage [7, 8]. Contributing to this therapeutic gap are non-specific clinical presentations that delay diagnosis, underutilization of quantitative imaging for valve and right ventricular assessment, and an absence of validated risk models tailored to the tricuspid population.

The introduction of transcatheter tricuspid therapies—pioneered by the TriClip system—has resulted in a paradigm shift for the treatment of TR. By adapting edge-to-edge repair principles from the mitral space, TriClip facilitates targeted leaflet coaptation via a minimally invasive, percutaneous approach. Early registry and trial data suggest robust reductions in regurgitant volume, symptomatic improvement, and favorable right ventricular remodeling [9]. With the evolution of the heart team concept, TriClip-mediated tricuspid transcatheter edge-to-edge repair (T-TEER) holds promise for high-risk surgical candidates and opens pathways for earlier intervention in less-advanced disease.

This manuscript provides a detailed, stepwise guide to tricuspid T-TEER with the TriClip device, integrating anatomical insights, imaging strategies, procedural nuances, and post-procedural management, designed to provide interventionalists with the knowledge to optimize outcomes for patients with significant TR.

## 2. Anatomical and Pathophysiological Considerations

**Fig. 1.**
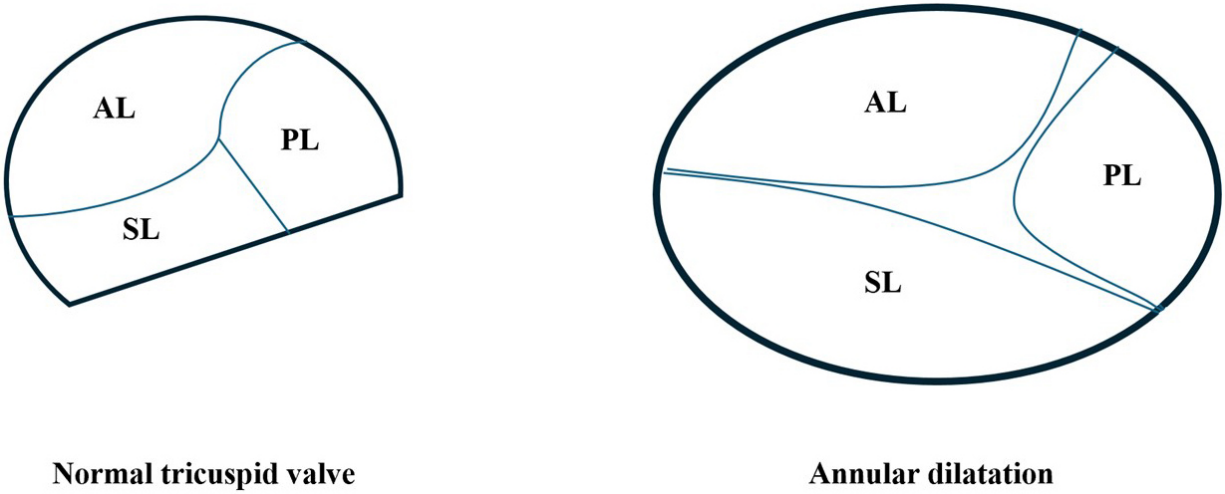
**Schematic representation of normal tricuspid valve anatomy and pathological annular dilatation in functional tricuspid regurgitation (TR)**. Left: Normal tricuspid valve configuration viewed from the right atrial perspective, showing the anterior leaflet (AL), posterior leaflet (PL), and septal leaflet (SL) coapting within a saddle-shaped, non-planar annulus. Right: In functional TR, chronic volume and/or pressure overload—typically due to right atrial enlargement or pulmonary hypertension—leads to annular dilatation, predominantly along the septal-lateral axis.

The tricuspid valve is a complex, nonplanar structure comprising three to five leaflets—most commonly anterior, posterior, and septal, but with frequent accessory leaflets—chordae tendineae, papillary muscles, and an annular ring supported by right ventricular geometry [10]. The anterior leaflet, by far the largest, and the posterior leaflet attach to distinct papillary muscles, whereas the septal leaflet, the smallest, anchors directly to the interventricular septum via chordae in the absence of a discrete papillary muscle (Fig. 1). This intricate apparatus lies in close proximity to the atrioventricular conduction system, and the saddle-shaped and crescentic geometry of the annulus makes it prone to dilatation under pressure or volume overload, leading to leaflet malcoaptation. Functional TR, which accounts for over 90% of cases, arises primarily from annular dilatation secondary to right atrial enlargement, pressure overload, or right ventricular dysfunction (Fig. 1). Common etiologies of TR include left-sided valvular disease, pulmonary hypertension, and chronic atrial fibrillation. Primary TR, which is less prevalent, arises from intrinsic leaflet abnormalities such as endocarditis, rheumatic and carcinoid disease. Regardless of the etiology, regurgitant volume overload leads to increased right atrial pressures, hepatic congestion, and progressive right ventricular (RV) dysfunction, which in turn exacerbates TR severity. Recognizing the potential for four or even five leaflets and the annulus’s dynamic morphology is critical when planning transcatheter edge-to-edge repair, since successful leaflet grasping and preservation of annular motion hinge on appreciating these variations.

## 3. Patient Selection and Pre-procedural Planning

### 3.1 T-TEER Candidates

Careful patient selection remains the cornerstone of successful T-TEER, and depends not only on the severity of regurgitation and patient comorbidities but also on the detailed leaflet and annular anatomy that determines procedural feasibility. Candidates for T-TEER are those who remain symptomatic—typically with exertional dyspnea, fatigue, hepatic congestion or peripheral edema corresponding to New York Heart Association (NYHA) class II–IV—despite optimized medical therapy including diuretics. High surgical risk, as determined by elevated European System for Cardiac Operative Risk Evaluation II (EUROSCORE II) or Society of Thoracic Surgeons (STS) risk scores, frequently tips the balance in favor of percutaneous repair. Many of these patients are octogenarians with prior left-sided valve interventions, atrial fibrillation, pulmonary hypertension, chronic kidney disease or hepatic dysfunction, all of which further increase operative risk. Quantitative echocardiographic criteria confirm the need for intervention: a vena contracta width of at least 7 mm, proximal isovelocity surface area for mitral regurgitation (PISA)-derived effective regurgitant orifice area ≥40 mm^2^ and regurgitant volume ≥45 mL indicate severe tricuspid regurgitation, while right ventricular contractile reserve—demonstrated by tricuspid annular plane systolic excursion (TAPSE) ≥13 mm and fractional area change ≥25%—identifies those most likely to derive benefit. Severely impaired RV function [TAPSE <10 mm or fractional area change (FAC) <20%] suggests limited potential for reverse remodeling [9, 11, 12, 13].

### 3.2 Transesophageal Echocardiography (TEE)

TEE remains the principal pre- and intra-procedural imaging modality for tricuspid TEER and is indispensable for anatomical assessment, procedural guidance, and immediate post-repair evaluation. Its role extends beyond device positioning to encompass detailed pre-procedural characterization of tricuspid valve morphology, right atrial and right ventricular remodeling, and quantification of TR severity.

A comprehensive pre-procedural TEE study systematically evaluates the number of leaflets, length, mobility, tethering, and calcification; annular size and dynamics; location and extent of coaptation gaps; and the presence of accessory leaflets or clefts. Pathological features such as rheumatic thickening, infective vegetations, or post-surgical repair materials are noted. Leaflet tethering angle and tenting height, measured in mid-systole, help predict technical feasibility and optimal clip selection. The severity of TR is quantified using a multiparametric approach (vena contracta width/area, PISA-derived effective regurgitant orifice area, regurgitant volume, hepatic vein flow reversal, continuous-wave Doppler density), realizing that eccentric jets and multiple regurgitant orifices may necessitate three-dimensional (3D) planimetry for accuracy.

### 3.3 Cardiac Computed Tomography Angiography (CCTA)

CCTA is a cornerstone modality in contemporary right-sided structural planning. When performed with prospective ECG-gating and a dedicated two-phase injection [arterial phase for coronary and right-heart anatomy; delayed venous phase for inferior vena cava (IVC)/superior vena cava (SVC)/hepatic venous mapping], CCTA provides high-fidelity 3D information that directly determines feasibility, device strategy, and procedural risk mitigation. Multiplanar and 3D reconstructions allow orthogonal septal–lateral and anteroposterior diameter measurements, annular perimeter/area, eccentricity index, and non-planarity angle. Quantification of leaflet tenting height/area and tethering vector orientation helps anticipate the need for wider clips, the optimal grasping pair (usually antero-septal vs. postero-septal), and whether residual TR is likely if tethering is extreme. CCTA can approximate coaptation gaps at mid-systole and identify commissural clefts/accessory scallops that complicate biplane guidance. In patients with poor echocardiographic windows, CT-derived annular sizing and RV/right atrium (RA) volumetry complement TEE to refine selection from the “favorable/feasible/unfavorable” anatomy categories defined in our review. Right atrial size, Eustachian ridge prominence, Chiari network, and RV trabeculations are delineated to anticipate catheter stability and chordal interactions. Systematic mapping of the IVC, SVC, and hepatic veins detects tortuosity, thrombus, filters, chronic occlusions, or variant drainage that may impact sheath support. This is particularly useful in very large right atria or tortuous IVCs where steerability and support are limited and in cases requiring a stiffer wire or different entry angle. CCTA accurately defines lead course relative to septal leaflet insertion and commissures, detects leaflet impingement or entrapment, and measures the distance/angle between the lead body and coaptation line. These data pre-empt single-leaflet device attachment and inform strategy (target a different leaflet pair, “clip-around” with caution, or involve EP for extraction/repositioning when lead adherence is present). Integration of CT-based lead mapping with 3D TEE improves targeting and minimizes the risk of entanglement.

### 3.4 Coronary Artery Disease (CAD) Assessment 

**Fig. 2.**
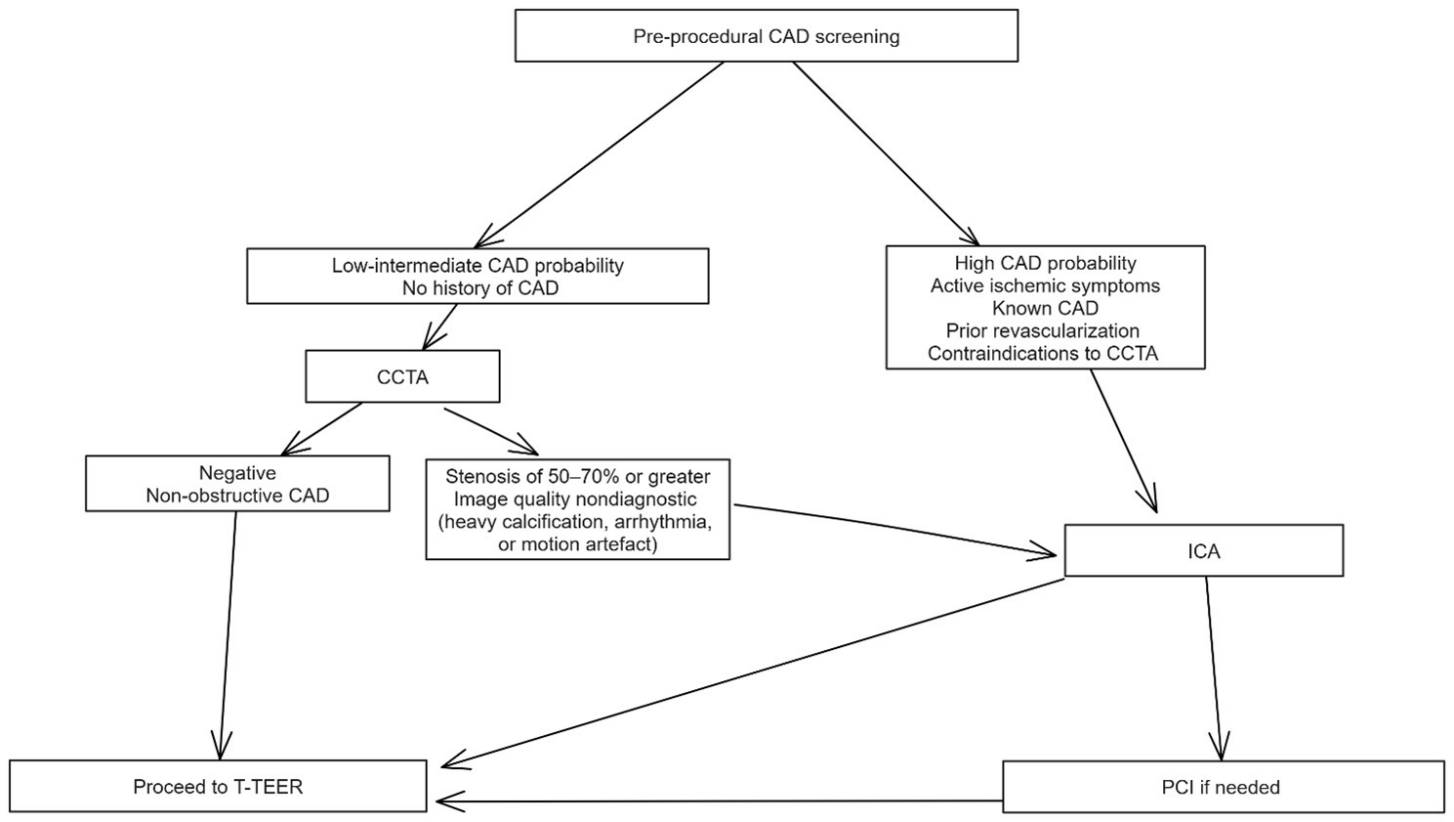
**Algorithm for pre-procedural coronary artery disease (CAD) screening in candidates for tricuspid transcatheter edge-to-edge repair (T-TEER)**. Patients are stratified by pre-test probability, history of CAD/revascularization, and feasibility of coronary computed tomography angiography (CCTA). Low-intermediate probability patients, with no prior CAD undergo CCTA as the initial test; negative or non-obstructive findings allow direct progression to T-TEER, whereas suspected ≥50–70% stenosis, or nondiagnostic image quality, prompts invasive coronary angiography (ICA). High probability patients, those with active ischemic symptoms, known CAD, prior revascularization or contraindications to CCTA, proceed directly to ICA. PCI should be performed for lesions deemed to be clinically significant. Abbreviations: PCI, percutaneous coronary intervention.

All candidates for T-TEER should undergo structured CAD screening within a probability- and history-based framework (Fig. 2). In patients with a low-to-intermediate pre-test probability of CAD or no history of coronary revascularization, CCTA serves as the preferred first-line investigation to exclude obstructive disease. A negative or non-obstructive CCTA allows direct progression to T-TEER. Conversely, if the study suggests a stenosis of 50–70% or greater, or if image quality is nondiagnostic due to heavy calcification, arrhythmia, or motion artefact, the patient should be referred for invasive coronary angiography (ICA).

Patients with a high pre-test probability of CAD, active ischemic symptoms, known CAD or prior revascularization, or contraindications to CCTA should proceed directly to ICA following a Heart Team discussion. In such cases, percutaneous coronary intervention (PCI) should be performed for lesions deemed to be clinically significant.

### 3.5 Anatomic Suitability

**Fig. 3.**
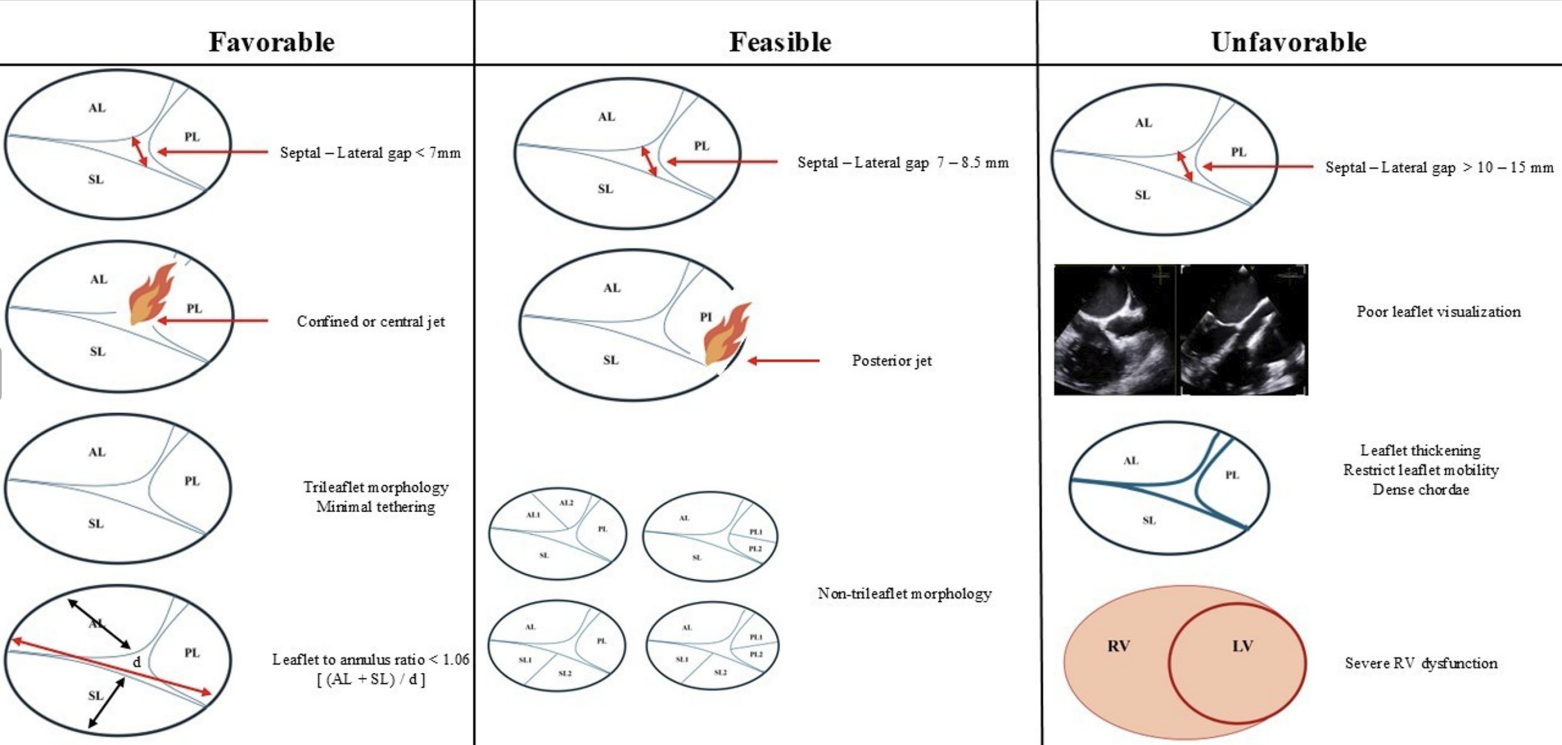
**Anatomical classification of tricuspid valve morphology for tricuspid transcatheter edge-to-edge repair (T-TEER): favorable, feasible, and unfavorable anatomies**. This schematic illustrates the key anatomical determinants guiding procedural feasibility and predicted outcomes in T-TEER using the TriClip system. Left column (Favorable anatomy): Septal–lateral coaptation gap <7 mm, central or confined tricuspid regurgitation (TR) jet, trileaflet configuration with minimal tethering, and a leaflet-to-annulus ratio <1.06 ([AL + SL] / annular diameter [d])—all of which predict successful and durable leaflet approximation. Middle column (Feasible anatomy): Intermediate findings, including coaptation gap 7–8.5 mm, posteriorly directed jet, non-trileaflet morphology (e.g., accessory leaflet subtypes), or moderate tethering, may allow adequate results but often require advanced imaging, precise clip orientation, and possible acceptance of residual mild TR. Right column (Unfavorable anatomy): Features such as coaptation gap >10–15 mm, poor leaflet visualization (e.g., due to acoustic shadowing or reverberation), leaflet thickening with restricted mobility or dense chordae, and severely impaired right ventricular (RV) function substantially reduce the likelihood of procedural success. These cases may be better suited to alternative therapies, including transcatheter valve replacement. Abbreviations: AL, anterior leaflet; PL, posterior leaflet; SL, septal leaflet; LV, left ventricular.

On the basis of these measurements, anatomy falls into one of three categories [14] (Fig. 3). “Favorable” anatomy represents a septal-lateral coaptation gap under 7 mm, confined or central jet location, trileaflet morphology with minimal tethering and a leaflet-to-annulus ratio below 1.06, all in the setting of low tethering height and right atrial volume, allowing straightforward septal–anterior or anteroseptal leaflet capture and durable reduction in regurgitation. “Feasible” anatomy—in which the coaptation gap measures between 7 and 8.5 mm or presents a posterior or non-trileaflet jet—may still yield satisfactory results but often requires meticulous clip orientation or acceptance of residual trace to mild regurgitation. An adequate grasp is possible if the risk of leaflet tear is low and right ventricular geometry remains favorable. In contrast, “unfavorable” anatomy—characterized by large coaptation gaps above 10–15 mm, dense chordae or tethering that severely restrict leaflet mobility, leaflet thickening, poor leaflet visualization, unfavorable device approach angles or severe right ventricular dysfunction—carries a high risk of leaflet detachment, single-leaflet device attachment or inadequate reduction of regurgitation, and may be better served by transcatheter valve replacement or alternative therapies.

A multidisciplinary heart team—including interventional cardiology, imaging specialists, cardiac surgery and anesthesia—must review each case in a pre-procedural conference to weigh symptomatic benefit, anatomical suitability and procedural risk. Preprocedural right heart catheterization may be useful in borderline cases for hemodynamic confirmation of RV function and pulmonary pressures. The discussion encompasses the choice of anesthesia, anticoagulation strategy, planning of vascular access and rehearsal of fluoroscopic and TEE co-registration. Comprehensive patient counseling sets realistic expectations: T-TEER aims to reduce rather than eliminate TR. While achieving moderate or less residual regurgitation at 30 days correlates with improved survival and quality of life, some patients—particularly those with “feasible” or “unfavorable” anatomy—may require multiple clips or consideration of transcatheter valve replacement to achieve optimal results [15, 16, 17]. By rigorously applying these selection and planning principles, operators can maximize procedural success, minimize complications and extend the benefits of transcatheter therapy to a patient population historically deemed inoperable.

## 4. Procedural Workflow

**Fig. 4.**
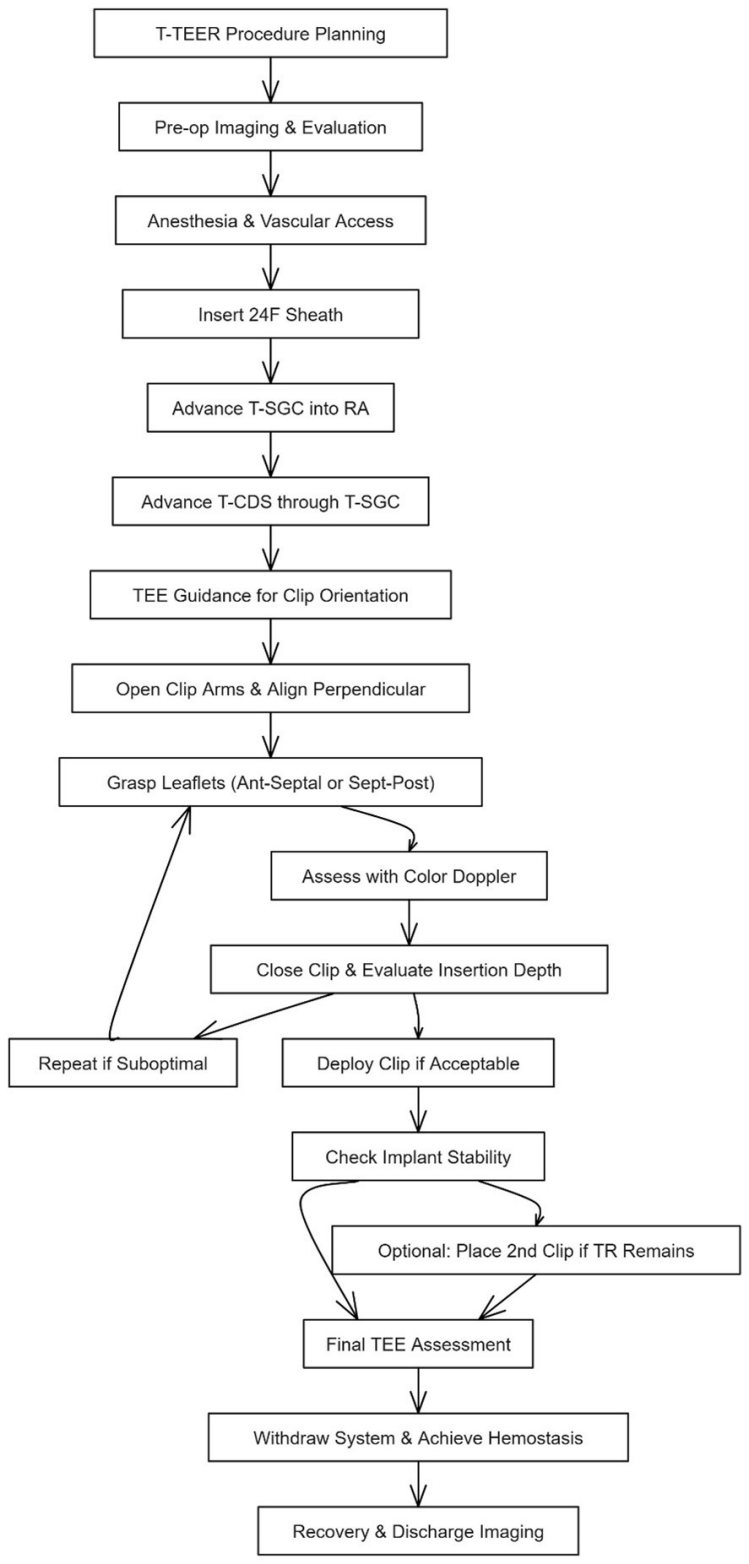
**Stepwise procedural workflow for tricuspid transcatheter edge-to-edge repair (T-TEER) using the TriClip system**. This diagram illustrates the standard procedural sequence for T-TEER with the TriClip device. After procedural planning and preoperative imaging, general anesthesia is administered and vascular access is obtained via the right femoral vein. A 24F sheath is inserted, followed by the TriClip steerable guide catheter (T-SGC) advanced into the right atrium (RA) and delivery system (T-CDS). Under real-time transesophageal echocardiography (TEE), the clip is aligned perpendicular to the coaptation line and leaflet grasping is performed—typically targeting the anterior-septal or septal-posterior leaflets. Leaflet insertion is assessed via TEE and color Doppler. If suboptimal, the grasp is repeated. Upon optimal capture, the clip is deployed, and implant stability is confirmed. A second clip may be implanted if significant residual regurgitation persists. The procedure concludes with final TEE assessment, system withdrawal, hemostasis, and post-procedural imaging prior to discharge. Abbreviations: TR, Tricuspid regurgitation; RA, right atrium.

**Fig. 5.**
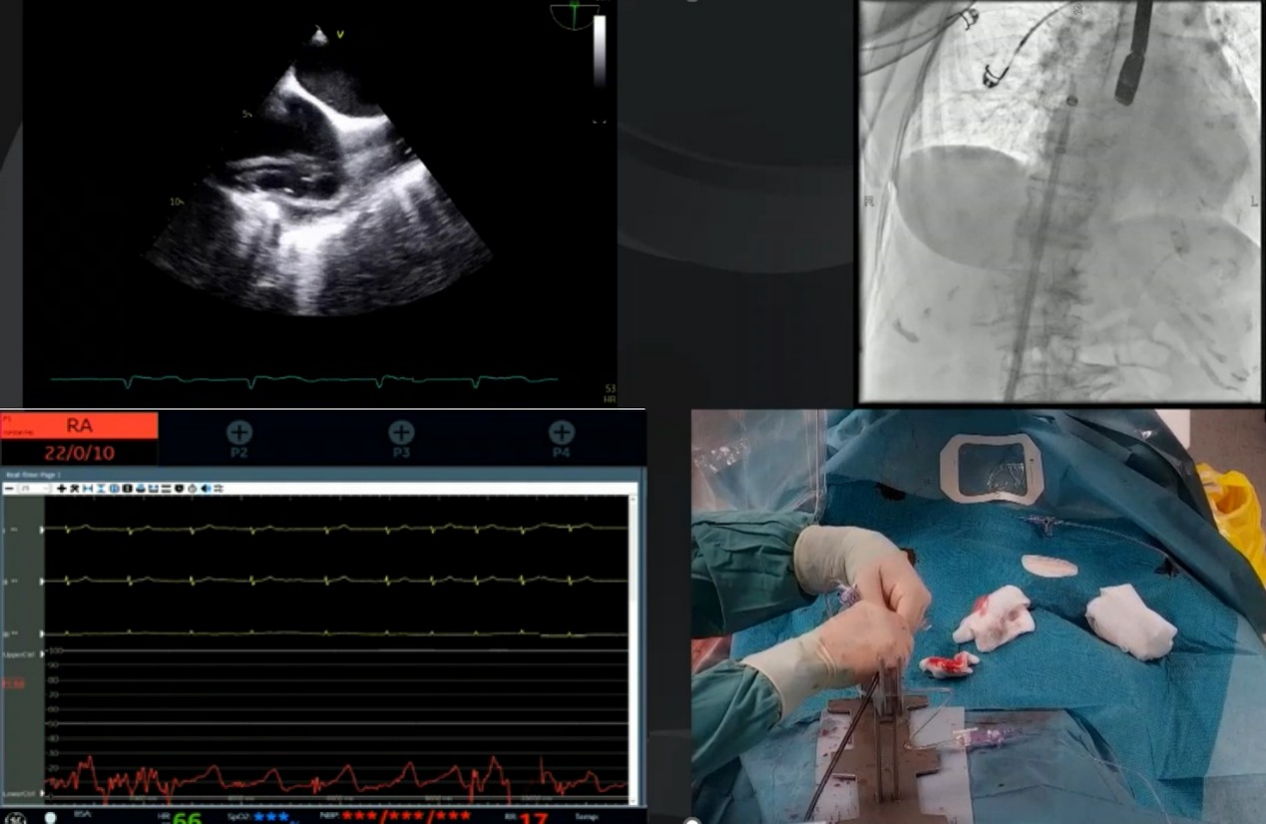
**Real-world procedural setup and imaging integration during TriClip tricuspid transcatheter edge-to-edge repair (T-TEER)**. Composite image from a live T-TEER procedure illustrating the multimodal integration required for optimal device guidance. Top right: Fluoroscopic imaging showing the 24F steerable guide catheter (T-SGC) advancing into the right atrium (RA). Top left: Transesophageal echocardiographic (TEE) showing the T-SGC entering the RA in real time, with the bicaval view (90–110 °C) used to guide safe advancement. Bottom right: Intraprocedural view of the hybrid lab setup, including vascular access management and T-SGC handling under sterile conditions. Bottom left: Hemodynamic monitoring with invasive RA pressure tracing.

Under general anesthesia, with the patient supine and routinely monitored (invasive arterial line, central venous pressure, full echocardiographic access), access is obtained through the right common femoral vein under ultrasound guidance, and a 24-French steerable sheath is advanced into the inferior vena cava over a stiff guidewire (Figs. 4,5). Systemic anticoagulation is initiated to maintain an activated clotting time above 250 seconds. The TriClip steerable guide catheter (T-SGC) is then advanced through the sheath directly into the right atrium under fluoroscopic guidance and real-time 3D TEE. The tricuspid clip delivery system (T-CDS) is then advanced through the guide and flexed down towards the tricuspid valve plane. Trajectory adjustments for coaxial alignment are performed by using the T-SGC steering knobs as required.

Under simultaneous 2D and real-time 3D TEE, the clip arms are gently opened, and the device is rotated until its arms lie perpendicular to the line of leaflet coaptation above the region of interest with the maximum tricuspid regurgitation. The perpendicularity is confirmed by both transgastric and multiplanar reconstruction imaging from mid-oesophageal views. These views also ensure that the clip is not inadvertently entangled in the chordae or right ventricular trabeculations.

When the operator is satisfied that the anterior and septal (or septal and posterior) leaflets are adequately positioned between the open arms, the clip is closed slowly while monitoring leaflet insertion depth—ideally achieving at least 5 mm of tissue capture per leaflet. Color Doppler on TEE immediately after closure quantifies residual regurgitant jets and assesses for new stenotic gradients; a mean tricuspid inflow gradient under 5 mmHg is considered to be acceptable. If leaflet grasp or residual TR is suboptimal, the arms are reopened, and the entire sequence of orientation and leaflet capture is repeated until ideal leaflet insertion and hemodynamic improvement are achieved.

Once satisfactory reduction in regurgitation and acceptable transvalvular gradients are confirmed, the clip’s locking mechanism is engaged, and the device is deployed from the delivery catheter. Stability of the implant is reconfirmed on 3D TEE to exclude single-leaflet device attachment. In cases where residual TR exceeds moderate severity, a second clip is ideally placed approximately 4–6 mm adjacent to the first; this process mirrors the initial steps but requires careful spatial planning to avoid interference between devices. Throughout the procedure, right atrial pressure tracings are observed for v-wave reduction, and intermittent heparin boluses may be administered to adjust anticoagulation. At completion, a comprehensive TEE assessment documents final TR grade, the tricuspid gradient, right ventricular size and function, and excludes a pericardial effusion. The steerable guide is then withdrawn, venous hemostasis is secured—typically with a figure of eight suture—and the patient is transferred to recovery for overnight monitoring, including a follow-up transthoracic echocardiogram prior to discharge.

Despite high procedural success rates, several technical challenges may arise during T-TEER. Table 1 (Ref. [18, 19, 20]) summarizes frequent pitfalls and recommended strategies to optimize outcomes.

**Table 1.** **Common technical challenges and troubleshooting strategies during TriClip T-TEER procedures**.

Challenge	Likely cause	Recommended solutions
Poor leaflet grasping [18]	Incomplete leaflet insertion, excessive tethering, or improper clip alignment	Reopen arms, optimize TEE angle, adjust SGC trajectory; ensure ≥5 mm insertion depth on both leaflets
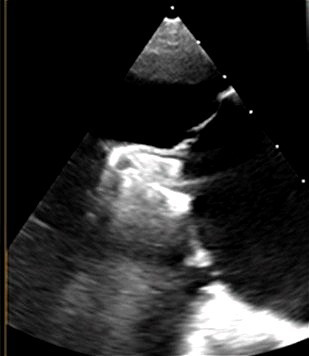		
Inability to achieve perpendicularity	Inadequate TEE visualization or limited guide catheter control	Use transgastric short-axis view for en face alignment; adjust flexion, rotation, and septal-lateral knobs
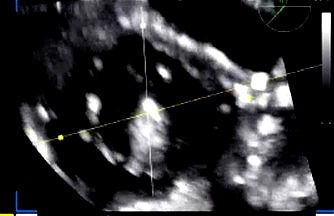		
Single leaflet device attachment (SLDA) [19]	Leaflet slippage or asymmetric grasping due to poor coaptation	Reposition and regrasp; consider using wider clip (XTW); re-evaluate leaflet anatomy before reattempting
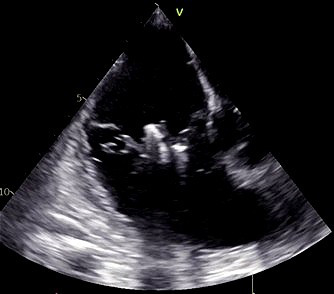		
Imaging artifacts obscuring coaptation zone	Reverberation from annular calcification or device shadowing	Switch to alternate TEE planes; use 3D or intracardiac echocardiography (ICE) as needed; reduce gain and optimize depth/focus settings
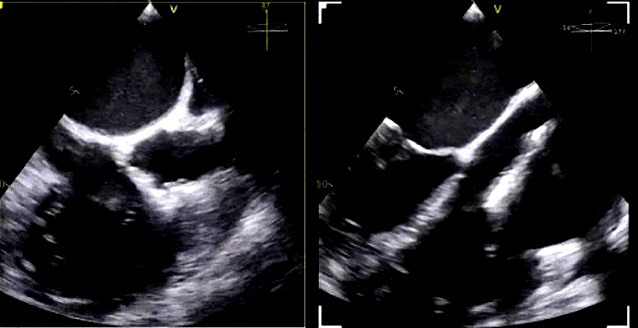		
Device entanglement with chordae or pacemaker lead [20]	Clip arm orientation or leaflet selection errors	Withdraw slightly and reposition under 3D/2D guidance; consider alternative grasp location
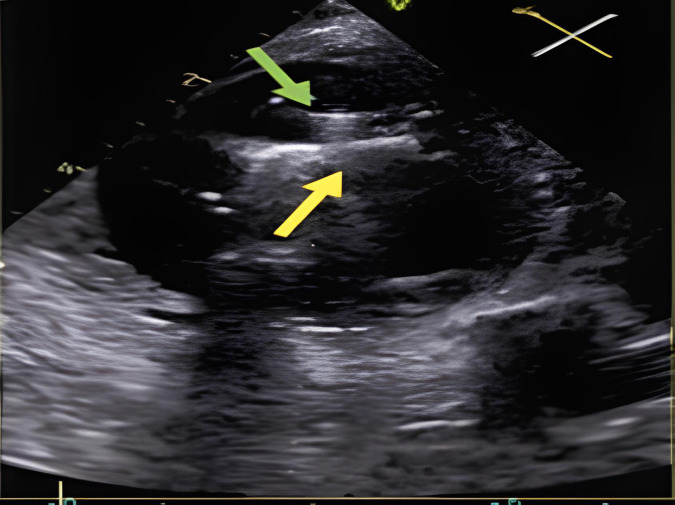		
Residual moderate-to-severe TR after 1st clip	Malcoaptation due to leaflet tethering, gap size, or eccentric jet	Consider second clip 4–6 mm adjacent; re-evaluate gap size; avoid excessive leaflet tension
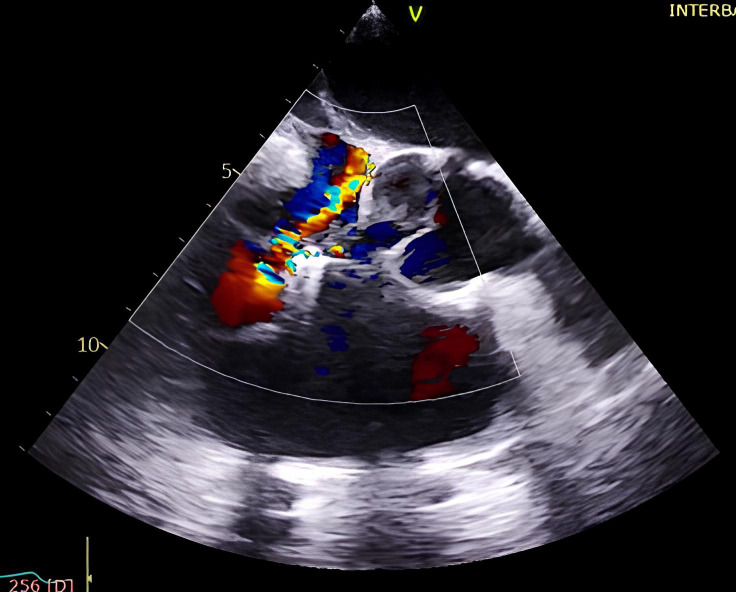		
Difficult navigation in large RA or tortuous IVC	Guide catheter instability or poor support	Use stiff wire for IVC support; apply slow, controlled torquing of the SGC
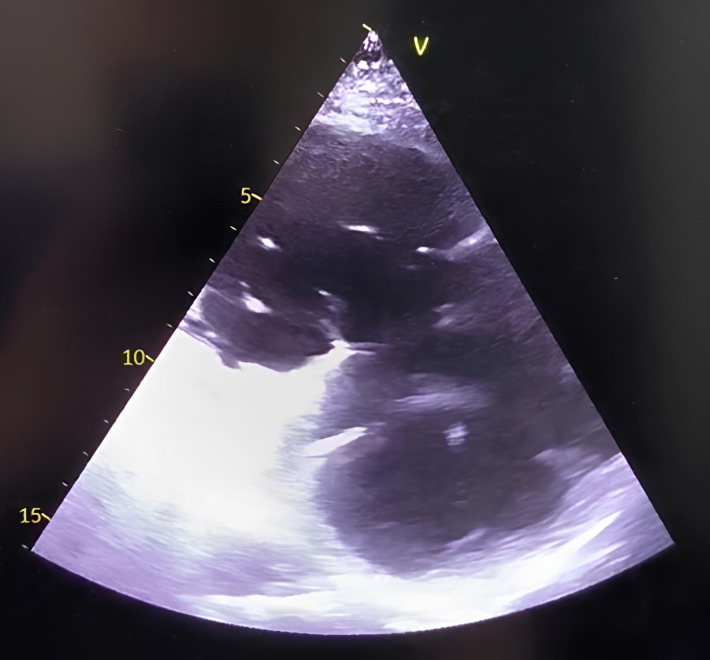		

Abbreviations: T-TEER, tricuspid transcatheter edge-to-edge repair; TEE, transesophageal echocardiographic; SGC, steerable guide catheter; TR, tricuspid regurgitation; RA, right atrium; IVC, inferior vena cava.

## 5. Intraprocedural Imaging Guidance During TriClip T-TEER

TEE plays an indispensable role in the execution of T-TEER with the TriClip system, providing real-time guidance for device navigation, leaflet orientation, grasping precision, and post-procedural assessment. A stepwise and anatomically informed imaging protocol is essential for procedural success, particularly given the complex and variable geometry of the tricuspid valve.

**Fig. 6.**
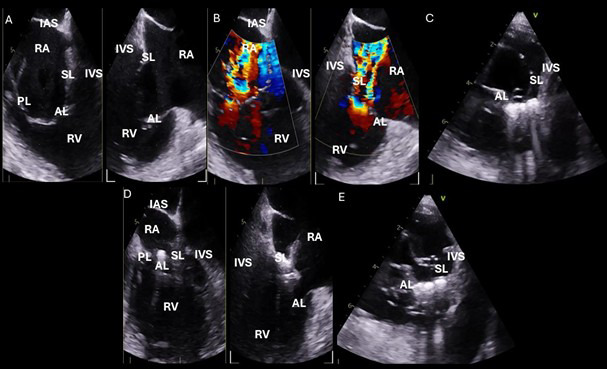
**Intraprocedural transesophageal echocardiographic (TEE) views during TriClip T-TEER**. (A) Biplane “commissural” tricuspid view (right ventricular inflow-outflow) at approximately 60 °C–90 °C with orthogonal plane at 0 °C–20 °C, used to guide clip trajectory toward the coaptation zone. (B) Color Doppler overlay in the same biplane view demonstrating a central tricuspid regurgitation jet. (C) Transgastric short-axis view (typically at 0 °C–30 °C), used to evaluate clip orientation and trajectory perpendicularity to the coaptation line. (D) Leaflet grasping attempt under real-time biplane TEE guidance, assessing adequate leaflet insertion and arm closure. (E) Confirmation of symmetric leaflet capture using the “papillon sign”—a bilobed fluttering of inserted leaflet tips visualized from the transgastric view, indicative of a successful grasp. Abbreviations: IAS, interatrial septum; RA, right atrium; IVS, interventricular septum; RV, right ventricle; SL, septal leaflet; AL, anterior leaflet; PL, posterior leaflet; T-TEER, tricuspid transcatheter edge-to-edge repair.

**Fig. 7.**
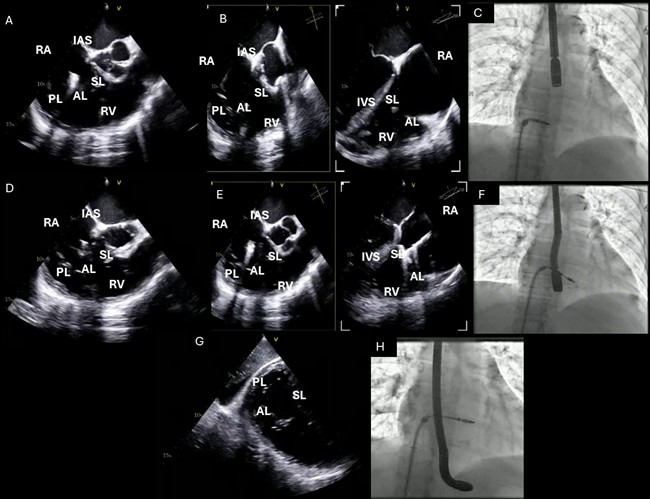
**TEE probe positioning and standard TEE views used during TriClip T-TEER**. (A–C): Mid-esophageal RV inflow-outflow view (commissural tricuspid view—obtained at approximately 60 °C to 90 °C); (D–F): Deep-esophageal RV inflow-outflow view; (G,H): Transgastric short-axis view of the TV (typically at 0 °C to 30 °C). Abbreviations: TV, tricuspid valve; IAS, interatrial septum; RA, right atrium; IVS, interventricular septum; RV, right ventricle; SL, septal leaflet; AL, anterior leaflet; PL, posterior leaflet; TEE, echocardiographic; T-TEER, tricuspid transcatheter edge-to-edge repair.

The imaging sequence begins with acquisition of the bicaval view, typically at a TEE angle between 90 °C and 110 °C, which allows continuous visualization of the superior and inferior vena cava and the right atrium [21, 22]. This view facilitates safe advancement of the T-SGC into the right atrium and ensures that the clip delivery system exits the sheath centrally and without entanglement in structures such as the atrial septum or Chiari network. Once the clip emerges from the catheter and is flexed downward toward the tricuspid annular plane, the focus shifts to a modified right ventricular inflow-outflow view—commonly referred to as the commissural tricuspid view—obtained at approximately 60 °C to 90 °C (Fig. 6A,B) [21, 22]. This view, used in conjunction with biplane imaging oriented orthogonally at 0 °C to 20 °C, allows real-time monitoring of the clip’s trajectory as it is advanced toward the targeted region of regurgitation. The biplane modality provides simultaneous insight into both septal-lateral and anterior-posterior positioning, which is critical for navigating to the zone of maximum regurgitant flow. Fig. 7 provides a detailed schematic of standard TEE views and corresponding fluoroscopic projections used for clip navigation and leaflet assessment.

Once the clip is positioned above the coaptation zone, the next objective is to optimize its orientation to be perpendicular relative to the valve’s coaptation line. This is accomplished by switching to transgastric short-axis imaging—typically at 0 °C to 30 °C—which offers an en face view of the tricuspid valve leaflets (Fig. 6C) [21, 22]. Additionally, three-dimensional multiplanar reconstruction (3D MPR) derived from commissural views (usually at 60 °C–90 °C) provides precise alignment data and allows for quantitative assessment and correction of the device’s rotational position, often referred to as “clocking” [21, 22]. Achieving near-perfect perpendicularity at this stage is critical for successful and symmetric leaflet grasping and minimizes the risk of single leaflet device attachment (SLDA).

When the clip trajectory is optimized, we return to the commissural view with biplane imaging to proceed with leaflet grasping (Fig. 6D) [21, 22]. After tentative closure, grasp confirmation is obtained using transgastric short-axis imaging. In this view, the characteristic “papillon sign”—a symmetric, bilobed fluttering pattern of the captured leaflets within the clip arms—serves as a visual surrogate for successful and balanced leaflet insertion (Fig. 6E) [21, 22]. Absence of this sign or evidence of asymmetric grasping necessitates reopening the clip and repeating the grasping maneuver under optimized imaging guidance.

Following definitive leaflet capture and clip deployment, a comprehensive final assessment is conducted to confirm procedural success. This includes color and continuous wave Doppler interrogation to evaluate residual tricuspid regurgitation. The mean tricuspid inflow gradient is measured and considered acceptable if it is less than 5 mmHg. Three-dimensional en face TEE imaging and deep esophageal or transgastric views are used to verify full leaflet coaptation and device stability. In anatomies with eccentric tethering or leaflet calcification, or in cases with limited acoustic windows, adjunctive intracardiac echocardiography (ICE) may be considered to improve anatomical resolution and guidance fidelity. Throughout the procedure, invasive right atrial pressure tracings are monitored for a reduction in v-wave amplitude, which provides additional hemodynamic confirmation of procedural efficacy.

This structured imaging strategy, incorporating sequential views with defined anatomical targets and angle-specific goals, enhances procedural reproducibility, minimizes complications, and is essential for the optimization of outcomes in T-TEER with the TriClip system.

## 6. Post-Procedural Management

Following T-TEER with the TriClip system, structured post-procedural management is essential to ensure optimal recovery, mitigate complications, and establish long-term benefit. Optimal post-procedural antithrombotic regimens remain empiric, as no dedicated randomized trials have addressed this population [23, 24]. In the absence of atrial fibrillation or other formal indications, most centers prescribe single antiplatelet therapy (aspirin 75–100 mg daily) for 3–6 months, although this practice varies [23, 24]. For patients with atrial fibrillation, anticoagulation (typically with direct oral anticoagulants or vitamin K antagonists) is resumed promptly after the procedure, assuming that vascular hemostasis is obtained [25].

A transthoracic echocardiogram (TTE) is routinely performed within 24 hours to evaluate clip position, residual TR severity, mean tricuspid inflow gradient, presence of a pericardial effusion, and right ventricular function [9, 11, 12, 13]. At 30 days, repeat echocardiography provides crucial data regarding early reverse remodeling and predicts functional response [9, 11, 12, 13].

Patients frequently require tailored diuretic regimens post-T-TEER, guided by daily weight, renal function, and symptomatology. Because reverse right heart remodeling may lag behind valvular correction, a cautious approach to volume depletion is advised. Loop diuretics are titrated to avoid intravascular depletion and renal injury, particularly in patients with pre-existing chronic kidney disease [9, 11, 12, 13].

All patients should undergo NYHA functional assessment at 30 days and 6 months, ideally with 6-minute walk distance testing and Kansas City Cardiomyopathy Questionnaire (KCCQ) scoring to document symptomatic function [9, 11, 12, 13]. Enrollment in a structured cardiac rehabilitation program is encouraged, particularly in frail or deconditioned individuals, to enhance physical recovery and quality of life.

Major procedural complications are uncommon but include access site bleeding, SLDA, device embolization, and new conduction abnormalities. Subclinical SLDA may evolve over weeks and should be excluded with serial imaging. New right bundle branch block or atrioventricular block may emerge, particularly in patients with pre-existing conduction delay, necessitating ECG monitoring and occasional pacemaker implantation [9, 11, 12, 13, 14].

Given the complex comorbidity profile of patients undergoing T-TEER, structured follow-up by a dedicated valve clinic or heart team is vital. Follow-up should include clinical evaluation, medication optimization (e.g., guideline-directed therapy for heart failure), rhythm surveillance, and reassessment of residual valve lesions or device integrity. In high-risk or anatomically borderline patients, early recognition of recurrent TR or an incomplete clinical response may prompt consideration of repeat intervention or transition to valve replacement strategies.

## 7. Comparative Insights: TriClip vs. PASCAL for T-TEER

While both TriClip (Abbott Vascular) and PASCAL (Edwards Lifesciences) systems have received CE Mark approval for transcatheter tricuspid repair and are based on edge-to-edge leaflet approximation, they differ in mechanical design and procedural handling (Table 2, Ref. [9, 12, 13, 16]). To date, no head-to-head randomized comparisons exist between the two systems.

**Table 2.** **Comparison between TriClip and PASCAL systems for tricuspid transcatheter edge-to-edge repair (T-TEER)**.

Feature	TriClip (Abbott Vascular)	PASCAL (Edwards Lifesciences)
Design origin	Derived from MitraClip, adapted for tricuspid use	Novel design, originally developed for mitral and tricuspid use
Leaflet grasping	Independent and staged grasping	Independent and staged grasping
Spacer	None	Central nitinol spacer for coaptation support
Clip sizes and configurations	NT, XT, NTW, XTW—variety of arm lengths and widths	PASCAL and PASCAL Ace—fixed sizes, less modular
Delivery system size	Tricuspid-specific steerable guide catheter (T-SGC)	Bulkier system, larger profile
Echocardiographic artifacts	Minimal to moderate, depending on anatomy	More frequent due to device bulk and spacer reflection
Manipulation and steerability	Tricuspid-specific design supports controlled RA navigation	Requires experience; manipulation may be more challenging in early use
Learning curve	Potentially shorter in centers with MitraClip experience due to platform familiarity	May require additional training due to unique design, spatial orientation, and staged clasping technique
Data support	TRILUMINATE trial, bRIGHT registry, and observational study [9, 13, 16]	CLASP TR [12]
Suitability for complex anatomy	Widely used in cases with pacing leads and variable annular morphology	Theoretically advantageous in large coaptation gaps or eccentric jets
Procedural time	Generally shorter	Often longer, particularly in early adoption phase
SLDA risk (single leaflet device attachment)	Lower when coaptation line is perpendicular and grasping is symmetric	Variable; may be higher in anatomies with poor visualization or asymmetric tethering

TriClip, adapted from the widely adopted MitraClip platform, has undergone specific adaptations for use in the tricuspid position, including a dedicated tricuspid steerable guide catheter and multiple clip sizes and widths (NT, XT, NTW, XTW). With the advent of the G4 platform, TriClip now includes independent and staged leaflet grasping, allowing the operator to optimize leaflet capture on one side before completing grasping on the other. In contrast, the PASCAL device features a central nitinol spacer intended to enhance coaptation and reduce tension on leaflets, particularly in patients with large coaptation gaps. It also allows staged leaflet capture, and its design aims to improve procedural efficacy in anatomies with severe leaflet tethering or eccentric jets. However, its larger profile and increased echogenic footprint may lead to more frequent imaging artifacts and longer procedural times, especially in centers early in their experience.

Both systems require familiarity with right-sided anatomy and imaging. TriClip may be preferred in patients with pacemaker leads or smaller right atria due to its tricuspid-specific catheter and longer clinical experience. PASCAL may be preferable in anatomies with extensive leaflet malcoaptation or larger coaptation gaps. However, both systems are effective when used in appropriately selected patients, and procedural success largely depends on anatomical suitability and center expertise.

The TriClip is supported by more extensive clinical data. The TRILUMINATE Pivotal trial [9] and its 2-year extension [16] provide robust, high-quality evidence for TR reduction, functional improvement, and safety. Conversely, PASCAL data, while promising [e.g., CLASP TR study [12]], are more limited in sample size and duration of follow-up. The rate of TR ≤ moderate at early follow-up appears comparable, but direct comparative analyses are lacking.

The learning curve associated with T-TEER varies between the TriClip and PASCAL systems and is influenced by device design, imaging complexity, and center experience. TriClip may offer a shorter learning curve in institutions with established experience in mitral TEER, given the platform’s direct lineage from the MitraClip and similarities in steering mechanics, clip deployment, and imaging workflows. This familiarity can facilitate earlier procedural reproducibility and broader adoption, particularly in programs already proficient with transseptal interventions. Nevertheless, both systems require specific training and an understanding of right-sided valve anatomy, and procedural success is ultimately determined by patient selection, operator experience, and institutional volume. Device choice should therefore be individualized, taking into account anatomical characteristics, technical requirements, and the learning environment of each center.

## 8. Combined Mitral and Tricuspid TEER: An Emerging Paradigm

In a growing population of patients presenting with combined mitral and tricuspid regurgitation, addressing both valves in a single procedure has emerged as a compelling therapeutic option. Recent studies have shown that persistent moderate-to-severe TR following isolated mitral TEER is associated with increased morbidity and mortality [26]. Therefore, simultaneous dual-valve repair may offer incremental clinical benefit over a staged or isolated approach [27, 28].

A novel strategy involving combined M-TEER and T-TEER using the same T-SGC has recently been evaluated in our center [29]. This combined procedure using a single T-SGC represents an off-label approach, although emerging data suggest its feasibility and safety. In a prospective cohort of 42 patients with advanced heart failure and dual-valve disease, combined repair with a single T-SGC demonstrated a 100% procedural success rate, with substantial reductions in mitral regurgitation (MR) and TR severity and mean procedural time of 70 minutes. At 1-year follow-up, all patients had NYHA class I–II status, 81% had trivial or mild TR, and heart failure hospitalizations were reduced to 7.1%, with no mortality. However, the study was from a single-center and lacked a comparator arm, limiting its generalizability.

This combined procedure is performed as follows. After obtaining ultrasound-guided femoral venous access, a transseptal puncture is performed according to standard practice, targeting a mid-to-inferior and posterior location. The T-SGC is then advanced into the left atrium, and one or more MitraClip G4 devices are implanted under real-time fluoroscopic and TEE guidance, following standard protocol. After successfully completing the M-TEER, the T-SGC is withdrawn into the right atrium. Subsequently, one or more TriClip G4 devices are implanted based on the patient’s anatomical requirements. Both the MitraClip and TriClip delivery systems are engaged with the T-SGC following the recommended alignment (“blue-to-blue” line), ensuring proper orientation. The term ‘blue-to-blue line’ refers to the visual alignment of the blue marker on the TriClip steerable guide catheter with the corresponding blue indicator on the clip delivery system. Ensuring this alignment prior to insertion confirms correct device orientation and clip arm opening in the intended anatomical plane, facilitating a perpendicular approach to the tricuspid coaptation line.

This single-system approach avoids the need for exchanging large-bore catheters, thereby minimizing vascular complications and shortening procedural time. The added septal-lateral steering knob of the T-SGC enables precise maneuverability for both valves, even in challenging anatomies, such as small left atria or low septal puncture sites.

This single-system approach remains investigational and should be considered off-label. Although early data from our center demonstrate procedural feasibility and encouraging outcomes, these results are derived from a single-center, non-randomized cohort. Larger multicenter studies are needed before this strategy can be recommended for widespread clinical use.

## 9. Clinical Outcomes and Evidence Synthesis

The emergence of T-TEER as a minimally invasive treatment for severe TR has been supported by accumulating evidence from both randomized trials and real-world registries. The TRILUMINATE Pivotal trial, the first randomized controlled study in this field, demonstrated that T-TEER was associated with a significant improvement in quality of life at one year, with a mean KCCQ improvement of 12.3 points and a high rate (87%) of TR reduction to moderate or less at 30 days [9]. These results were achieved with a favorable safety profile, with 98.3% of patients free from major adverse clinical events at 30 days. Although rates of death and heart failure hospitalization did not differ significantly between groups at 1 year, the hierarchical composite primary endpoint significantly favored TEER over medical therapy, underscoring a meaningful patient-centered benefit. The 2-year results from the TRILUMINATE single-arm cohort further demonstrated the durability of repair, with ≥1-grade TR reduction in ∼85% of patients and ≤moderate TR in 60%, accompanied by sustained improvements in NYHA class, 6-minute walk distance, and quality of life. Importantly, all-cause hospitalizations decreased by ∼49% compared with the pre-intervention year, highlighting the procedure’s long-term clinical impact [16].

The TRI.FR trial, a randomized study from France and Belgium, provided additional validation of T-TEER’s efficacy [11]. At one year, the composite clinical endpoint (including NYHA class, patient global assessment, and major cardiovascular events) was improved in 74.1% of patients treated with T-TEER plus medical therapy compared to 40.6% of those treated with medical therapy alone. Additionally, TR severity was significantly lower in the interventional arm, with only 6.8% of patients retaining massive or torrential TR.

While these studies confirm the safety and efficacy of T-TEER in selected populations, it is important to acknowledge their limitations. The TRILUMINATE [9] and TRI.FR [11] trials excluded patients with severe pulmonary hypertension (RVSP >60 mm Hg), persistent or longstanding atrial fibrillation (>6 months), and significant hepatic or renal dysfunction. These exclusions limit direct extrapolation to the broader real-world cohort, many of whom present with such comorbidities. Thus, careful individualized patient selection remains essential in clinical decision-making, particularly in those beyond the inclusion criteria of existing trials.

Real-world data from the bRIGHT registry support the reproducibility of trial results [13]. Among 511 patients treated with TriClip, TR was reduced to moderate or less in 81% of patients at one year, accompanied by a 19-point increase in KCCQ and a 75% improvement in NYHA class. These functional and symptomatic benefits were most pronounced in patients who achieved significant TR reduction at 30 days. Importantly, TAPSE and baseline renal function were identified as independent predictors of 1-year mortality.

Notably, the recently released 2025 ESC/EACTS Guidelines for the Management of Valvular Heart Disease for the first time formally recommend T-TEER in symptomatic patients with severe TR who are at high or prohibitive surgical risk, provided anatomy is suitable and procedures are performed in experienced Heart Valve centers [25]. This represents a major shift in guideline-based practice, aligning contemporary evidence with clinical decision-making and positioning TEER as a guideline-endorsed therapeutic option for patients who previously had few viable treatments.

## 10. Future Implications

These trials and registries establish T-TEER as a safe, effective, and durable intervention for patients with severe TR and prohibitive surgical risk. The consistent improvements in quality of life, functional status, and right heart reverse remodeling across studies underline the clinical importance of reducing the severity of TR. Importantly, the extent of residual TR post-procedure correlates with outcomes, highlighting the need for optimized patient selection and procedural technique.

As technology evolves, patient-specific strategies will become increasingly refined. The future landscape may include device fusion platforms, annular reduction therapies, and dedicated imaging tools for tricuspid disease. Multimodal imaging and computational modeling may further enhance anatomical selection and procedural planning. Comparative trials between repair and replacement, as well as trials assessing earlier intervention in moderate TR or asymptomatic patients, are needed. Additionally, longer-term follow-up will be critical to establish durability, impact on survival, and potential disease-modifying effects.

Despite promising clinical results, the widespread adoption of T-TEER faces significant logistical and economic barriers. The TriClip device is associated with considerable procedural costs, often several-fold higher than those of optimized medical therapy. The need for high-level intraprocedural TEE, hybrid interventional-imaging laboratories, and experienced multidisciplinary teams further limits its accessibility, especially in low-volume or resource-limited centers. Future cost-effectiveness analyses and health policy adaptations will be essential for equitable dissemination of T-TEER technologies.

## 11. Conclusions

T-TEER using the TriClip system has emerged as a viable and increasingly validated treatment for patients with severe tricuspid regurgitation who are at high or prohibitive surgical risk. This step-by-step review underscores the critical role of anatomical assessment, imaging-guided device navigation, and procedural optimization in achieving successful outcomes. Comparative insights with the PASCAL system, as well as the evolving application of combined mitral and tricuspid repair using a single platform, reflect the expanding potential of edge-to-edge techniques in complex valve disease. As clinical experience and device technologies continue to evolve, T-TEER is poised to become a cornerstone in the management of functional tricuspid regurgitation, addressing a long-standing therapeutic gap.
